# A commentary on ‘Robotic-assisted total knee arthroplasty is more advantageous for knees with severe deformity: a randomized controlled trial study design’

**DOI:** 10.1097/JS9.0000000000000903

**Published:** 2023-11-21

**Authors:** Chenzhong Zong, Qingfu Wang, Gao Kuo, Xueqi Dong, Xiaoqing Zhang

**Affiliations:** a Beijing University of Traditional Chinese Medicine; b Department of Tendons and Traumatology, Third Affiliated Hospital of Beijing University of Traditional Chinese Medicine; c China Health Media Group, Beijing, People’s Republic of China

*Dear Editor*,

Robotic-assisted total knee arthroplasty (TKA) systems, which can effectively increase prosthesis placement accuracy and potentially improve limb axis alignment, are used to address TKA problems^1,2^. Tian *et al*.^3^ conducted a multicenter, prospective, randomized controlled study to analyze patients with several deformities on knees who received a new robotic-assisted TKA system. To summarize, the robotic-assisted TKA system is a safe and effective TKA system that can be used routinely in patients with severe preoperative lower extremity alignment deviations.

A total of 144 patients who underwent TKA between June 2021 and December 2021 were analyzed. Two groups of patients were randomly assigned, with 72 undergoing robotic-assisted TKA and the remaining 72 undergoing traditional TKA. Only patients with a preoperative hip–knee–ankle (HKA) angle greater than or equal to 6° had intergroup significant differences in the rate of CFCA (coronal femoral component alignment) outliers and CFCA deviation, while there was no difference in slight preoperative lower extremity alignment deviations. It is proposed that a robotic-assisted TKA system has advantages in adjusting the prosthesis position for patients with severe preoperative lower extremity alignment deviations.

The research also sheds light on the radiographic parameters, surgical time, and demographic information that may affect the postoperative HKA angle. According to the results of multiple linear regression analysis, the preoperative HKA angle deviation would affect the recovery of the postoperative HKA angle deviation. Despite the fact that the results confirmed that robotic-assisted surgery (RAS) was more effective than conventional surgery (CON) for patients with severe preoperative lower extremity alignment deviations, there were still many limitations. First, the sample size was small, and this study only followed patients for a short period of time, so the long-term effect of RAS is unknown. Second, surgeons in the RAS group will inevitably operate more carefully, which may lead to bias. Finally, the bias exists due to the surgeons’ high level of skill.

Extensive multicenter studies are required to validate this finding. Determining the best course of treatment also requires more thorough prospective studies, clinical trials, and investigation of indications and risk factors in various patient subgroups. The following factors can be taken into account when planning future research projects: First, a large-scale multicenter study should be conducted to validate current findings and to further investigate indications and risk factors in different patient subgroups. Second, based on the patient’s preoperative HKA angle and other prognostic factors, a de-escalation strategy can be used to individually decide whether to use robotic-assisted TKA during clinical trial design. Finally, more research is needed to determine whether RAS has better long-term radiographic and clinical outcomes than traditional surgical techniques^4,5^.

This study provides insight into the short-term effect of a new robotic-assisted TKA system. The implications of this issue for clinical practice are immense. This multicenter, prospective, randomized controlled study provides preliminary evidence that the robotic-assisted TKA system is a safe and effective system for TKA and lays the groundwork for larger, multicenter studies. The factors that could influence the HKA angle after surgery are also discussed. This proactive thinking offers helpful direction and motivation for the further advancement of this field of study. To summarize, this study offers preliminary evidence that the robotic-assisted TKA system is a safe and effective method for TKA and directs the course of future research in this area. Through further exploration and research, the long-term radiographic and clinical outcomes of the robotic-assisted TKA system can be obtained.

## Ethical approval

This manuscript is a commentary and does not require ethical approval.

## Consent

This manuscript is a commentary and does not need patient consent.

## Sources of funding

This manuscript is a commentary without any sources of funding.

## Author contribution

C.Z. and Q.W.: study concept or design, data collection, data analysis or interpretation, and writing the paper; G.K. and X.D.: data collection; X.Z.: study concept or design, writing, and revising the paper.

## Conflicts of interest disclosure

This manuscript is a commentary without any conflicts of interest.

## Research registration unique identifying number (UIN)

This manuscript is a commentary and does not require any UIN.

## Guarantor

Xiaoqing Zhang.

## Data availability statement

This manuscript is a commentary and does not require a data availability statement. However, all the data from the current study are publicly available.

## Provenance and peer review

This manuscript is a comment without being invited.
